# SNRPB promotes gastric cancer progression by regulating aberrant splicing of PUF60

**DOI:** 10.1038/s41419-025-08011-2

**Published:** 2025-10-07

**Authors:** Dan Xiang, Jiaxin Yang, Miaofang Xiao, Cong Long, Yangxuan Lin, Chenchen Mao, Xin Liu, Dianfeng Mei, Wangkai Xie, Zheng Han, Chenbin Chen, Xiaoming Lin, Xian Shen, Xiangyang Xue, Tanzhou Chen

**Affiliations:** 1 Wenzhou Collaborative Innovation Center of Gastrointestinal Cancer in Basic Research and Precision Medicine, Wenzhou Key Laboratory of Cancer-related Pathogens and Immunity, Experiemtial Center of Basic Medicine, Department of Microbiology and Immunology, Institute of Molecular Virology and Immunology, School of Basic Medical Sciences, Wenzhou Medical University, Wenzhou, China; 2 Department of Gastrointestinal Surgery, Taizhou Hospital of Zhejiang Province affiliated to Wenzhou Medical University, Taizhou, China; 3 GMU-GIBH Joint School of Life Sciences, The Guangdong-Hong Kong-Macao Joint Laboratory for Cell Fate Regulation and Diseases, Guangzhou Medical University, Guangzhou City, China; 4 Hantai District Maternal and Child Health Hospital, Dongguan, China; 5 Department of Thoracic Surgery, The First Affiliated Hospital of Wenzhou Medical University, Wenzhou, China; 6 Department of General Surgery, The Second Affiliated Hospital and Yuying Children’s Hospital of Wenzhou Medical University, Wenzhou, China; 7 Department of Clinical Laboratory, Xuyong County People’s Hospital, Luzhou City, China; 8 Department of General Surgery, The First Affiliated Hospital of Wenzhou Medical University, Wenzhou, China; 9 The Department of Gastroenterology and Hepatology, The First Affiliated Hospital of Wenzhou Medical University, Wenzhou, P.R. China

**Keywords:** Gastric cancer, Tumour biomarkers

## Abstract

Alternative splicing is a pivotal regulatory mechanism in cellular biology that critically influences the tumorigenesis, progression, and phenotypic diversity of cancer. This study aimed to assess the intricate details and regulatory mechanisms of alternative splicing in gastric cancer. We constructed a comprehensive map of aberrant alternative splicing events in gastric cancer through bioinformatic analysis of public databases and clinical samples. Our study identified many abnormal splicing events in gastric cancer tissues, with exon skipping being the most frequent event. SNRPB, a key spliceosome component and principal splicing factor, was associated with the aberrant splicing of numerous splicing factors and oncogenes, influencing the p53 signaling pathway in the development and progression of gastric cancer. SNRPB directly regulates the selective splicing of TP53 by modulating its downstream factor, PUF60, thus facilitating the initiation and progression of gastric cancer. Therefore, SNRPB overexpression is linked to poor prognosis in gastric cancer and is a potential biomarker and therapeutic target.

## Introduction

Gastric cancer (GC) is a malignant tumor with a high morbidity and mortality rate worldwide [1], with a 5-year overall survival rate of <40% [2]. Patients with early GC can achieve an ideal prognosis through endoscopic resection and gastrectomy [3, 4]; however, most patients with GC are already at an advanced stage at the time of diagnosis. The molecular mechanisms underlying GC are complex, and its clinical manifestations are diverse. In the past, numerous studies have focused on understanding the occurrence and development of GC through the examination of copy number variations and aberrant gene expression profiles. However, relatively less attention has been given to a crucial aspect of gene expression regulation: alternative splicing (AS) [5]. The existing studies have shown that certain splicing events may promote the proliferation and invasion of GC cells by affecting the expression of cell cycle regulators or cell adhesion molecules [6, 7]. Additionally, the microenvironment of GC cells, including tumor stromal cells, extracellular matrix, and secreted factors, may further promote tumor development and metastasis by influencing the pattern of AS [8, 9]. Therefore, elucidating the characteristics of AS in GC and its regulatory mechanisms has important clinical value for the early diagnosis, precision treatment, and prognostic evaluation of GC [10].

Among the critical regulators of splicing fidelity are the spliceosome and auxiliary splicing factors. Small nuclear ribonucleoprotein polypeptide B and b1 (SNRPB) is a crucial core component of the spliceosome, playing a vital role in recognizing splice sites and assembling the splicing machinery during both constitutive splicing and AS. Reduced expression of SNRPB leads to a decrease in the expression levels of small nuclear ribonucleoproteins (snRNPs) and significantly reduces the expression levels of many additional variable exons. These exons primarily function in RNA processing and RNA binding [11]. Some current researches show that SNRPB may also play an important role in the progression and promotion of cancer, such as glioblastoma [11], non-small cell lung cancer [12, 13], hepatocellular carcinoma [14, 15], and cervical cancer [16]. However, the role of SNRPB in GC is unclear.

Alongside core components, auxiliary splicing factors such as Poly(U)-Binding Splicing Factor 60 (PUF60) play pivotal roles in fine-tuning AS decisions. PUF60, also known as FIR, Hfp, or Ro-bp1, is initially identified as a 60 kDa splicing factor that binds to poly-U and is essential for the efficient splicing of multiple introns [17]. It is a member of the U2 small nuclear ribonucleoprotein accessory factor (U2AF) family, homologous to U2AF65, and functions in conjunction with U2AF65 to bind to the 3’ splice site. This interaction regulates splicing efficiency and influences the type of splicing. [18]. The past studies have demonstrated that the expression level of PUF60 is abnormally elevated in various cancers [19, 20]. For instance, in bladder cancer, high levels of PUF60 expression are associated with a malignant phenotype [21]. However, some research indicates that it is not the overall expression level of PUF60 that drives cancer development, but rather the expression levels of different PUF60 transcripts. For example, in colorectal cancer, the alternative splice variant of PUF60, FIRΔexon2 (which lacks the amino terminus of exon 2), is absent in paracancerous tissues and can elevate the level of c-myc, thereby enhancing the tumor’s anti-apoptotic capabilities [22]. Unfortunately, the specific role of PUF60 in GC has yet to be fully elucidated.

In this comprehensive study, we meticulously examined the landscape of AS events in GC and identified a group of key splicing factors that exhibited abnormal expression patterns in the context of the disease. Utilizing a multifaceted approach, we investigated the complex mechanisms of the key splicing factor SNRPB and its downstream splicing factor PUF60 in the occurrence and progression of GC. Our research is anticipated to significantly impact the field by uncovering new intervention targets, ultimately leading to more effective treatment options for patients with GC.

## Results

### Aberrant AS events in GC

**Fig. 1:**
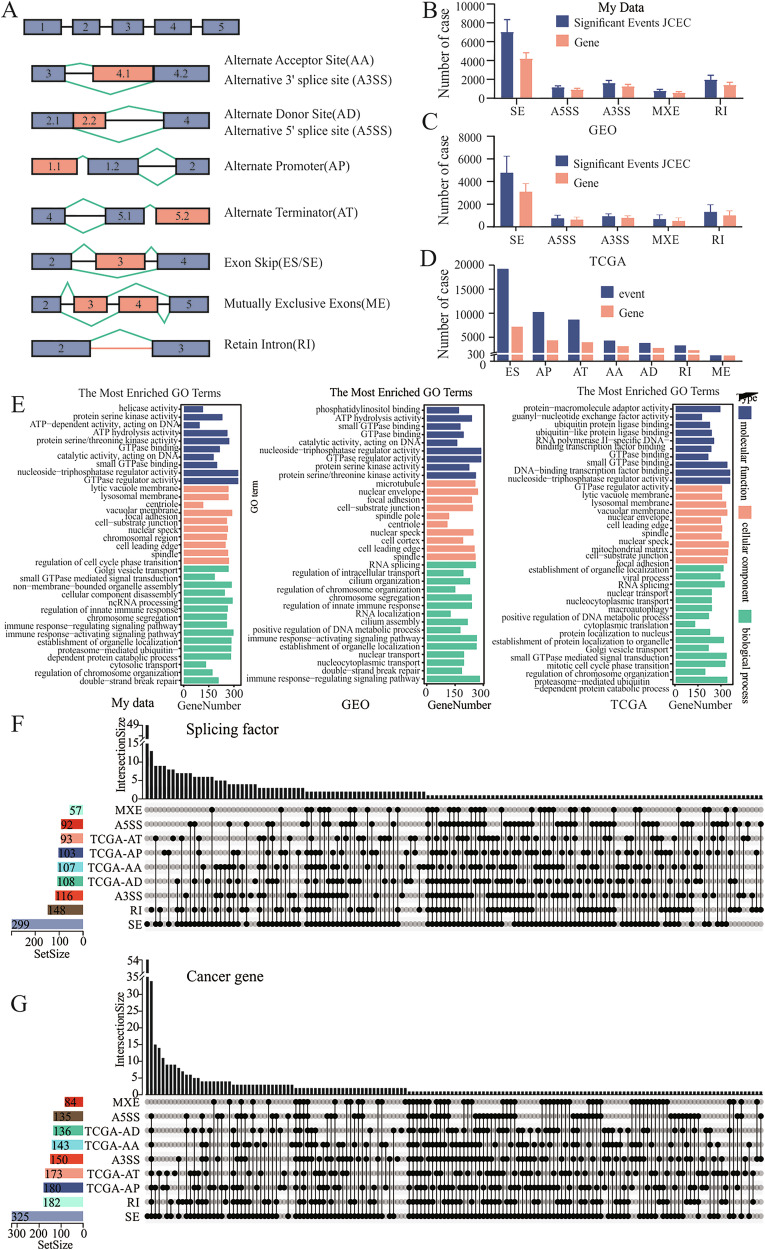
The aberrant splicing events are observed in gastric cancer. **A** Types of alternative splicing. **B**–**D** The occurrence of abnormal alternative splicing events in gastric cancer in the samples submitted for testing (**B**), GEO dataset (GSE172032) (**C**), and TCGA database (**D**). **E** Gene Ontology (GO) enrichment analysis of genes with abnormal alternative splicing events. **F** UpSet diagram illustrating the number and event types of splicing factors with aberrant splicing in gastric cancer. **G** UpSet diagram illustrating the number and event types of the oncogenes with aberrant splicing in gastric cancer.

We outlined the comprehensive landscape of abnormal AS events in GC. AS has nine splicing modes (Fig. 1A) [23]. The most common abnormal splicing event in GC was exon skipping (ES/SE), and the least common was mutually exclusive exon (MXE/ME) (Fig. 1B). This is consistent with the results of The Cancer Genome Atlas (TCGA) and Gene Expression Omnibus (GEO) datasets (GSE172032) (Fig. 1C, D). We conducted Gene Ontology enrichment analysis on genes exhibiting abnormal splicing events and compared the in-house data with findings from public databases (Fig. 1E). These results indicated that, in terms of molecular function, genes with abnormal splicing events were closely related to GTPase regulation. These genes were enriched in RNA splicing and the biological processes involved in controlling the internal structure and function of cells.

We then summarized the 378 splicing factors that underwent aberrant splicing events and created an UpSet graph for visualization (Fig. 1F). Among them, 56 splicing factors occurred only in ES events, and 13 splicing factors occurred only in retained intron (RI) events. Most splicing factors undergo at least two events. We also analyzed the differential expression of these genes in each dataset (Fig. S1). Additionally, we summarized and visualized 390 cancer genes with splicing events (Fig. 1G). Among them, 52 cancer genes occurred only during ES events, and seven occurred only during RI events.

Similarly, we performed a differential analysis of these 390 cancer genes (Fig. S2). A subset of these oncogenes stands out because of their dual aberrations; they are differentially expressed across various conditions and manifest at least three irregular splicing events. This dual anomaly highlights their potential role in the complex molecular landscape of cancer.

### SNRPB may be a core splicing factor in GC

**Fig. 2:**
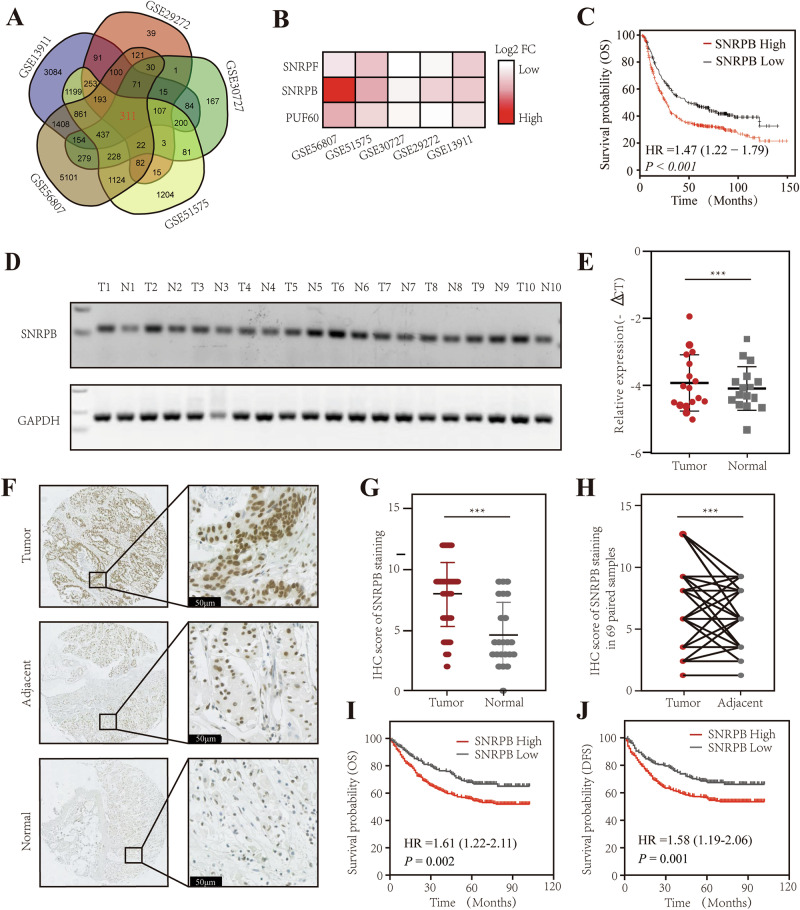
The splicing factor SNRPB is overexpressed in gastric cancer and associated with a poor prognosis. **A** Venn diagram illustrating the intersection of differentially expressed genes between cancer and adjacent tissues in five gastric cancer datasets: GSE13911, GSE29272, GSE30727, GSE51575, and GSE56807. **B** The expression levels of SNRPB, SNRPF, and PUF60 in five datasets. **C** The Kaplan–Meier analysis evaluated the overall survival (OS) of gastric cancer patients in the SNRPB high- and low-expression groups in the GEO database by the R2 tool (http://r2.amc.nl). **D** The RNA expression levels of SNRPB in 10 pairs of gastric cancer and adjacent tissues were detected using PCR. T stands for tumor, and N stands for adjacent tissues. **E** The RNA expression levels of SNRPB in 17 pairs of gastric cancer and adjacent tissues were detected using qPCR. **F** Representative immunohistochemical images of normal gastric mucosal tissue from weight-loss patients, gastric cancer patients, and paired normal tissues adjacent to the cancer. **G**, **H** The immunohistochemical scoring was conducted on 23 normal weight-loss patients’ gastric mucosal tissues, 69 paired gastric cancer tissues, and corresponding adjacent normal tissues. **I**, **J** The Kaplan–Meier analysis was conducted on the high- and low-expression groups of SNRPB protein to assess patients’ overall survival (OS) and disease-free survival (DFS). *P* < 0.05*, *P* < 0.01**, *P* < 0.001***.

In the preceding discussion on the landscape of AS in GC, we observed that certain splicing factors exhibited both AS and differential expression, which may have significantly contributed to the abnormal AS events observed in GC (Fig. S1). Consequently, we conducted a computational analysis of the five GEO datasets and identified 311 differentially expressed genes (Fig. 2A). Among these, three splicing factors, SNRPB, SNRPF, and PUF60, were overexpressed and exhibited abnormal splicing patterns in GC (Fig. 2B). Notably, the differential expression of SNRPB was more pronounced than that of the other two genes, and survival analysis indicated that patients with high SNRPB expression had a poorer prognosis (Fig. 2C).

### SNRPB is overexpressed in GC and is associated with a poor prognosis

The mRNA expression of SNRPB was assessed in 10 paired GC and normal tissues, revealing high expression of SNRPB in GC tissues (Fig. 2D, E). Immunohistochemical staining (IHC) showed that SNRPB was localized to the nuclei of cancer cells (Fig. 2F). Furthermore, SNRPB expression was found to be elevated in tumor tissues compared to that in adjacent normal tissues (Fig. 2G, H). Subsequently, based on the diverse H-scores obtained from IHC, patients were categorized into low and high SNRPB expression groups. High SNRPB expression was significantly associated with serum carcinoembryonic antigen (CEA) levels, tumor length, degree of tumor differentiation, depth of invasion, lymph node metastasis, and tumor node metastasis (TNM) staging of the tumor (*P* < 0.01) (Table 1). Additionally, Kaplan–Meier survival analysis demonstrated that patients with high SNRPB expression had a poorer prognosis in terms of overall survival (OS) (*P* = 0.002, Fig. 2I) and disease-free survival (*P* = 0.001, Fig. 2J) than those with low SNRPB expression; this aligns with findings from public databases (Fig. 2C).

**Table 1:** The differences in clinical characteristics between the high-expression SNRPB group and the low-expression SNRPB group.

Variables	Low expression	High expression	*n*	*X*^2^	*P*^a^ value
Numbers	215	486			
Age				1.959	0.162
<60	119	239	358		
≥60	94	238	332		
Gender				1.323	0.250
Male	160	341	501		
Female	55	145	200		
Adjuvant chemotherapy				2.586	0.108
No	74	137	211		
Yes	141	346	487		
Serum CEA (ng/mL)				7.206	**0.007**
<5	175	361	536		
≥5	26	101	127		
Serum CA199 (U/mL)				2.988	0.084
<37	166	368	534		
≥37	23	79	102		
Diameter (cm)				9.941	**0.002**
<4	116	200	316		
≥4	98	284	382		
Lauren type				0.050	0.824
Intestinal type	102	235	337		
Diffuse type	113	251	364		
Grade				10.407	**0.008**
Poorly and undifferentiated G3	144	313	457		
Moderately G2	57	15	222		
Well G1	10	6	16		
Depth of invasion				26.840	**<0.001**
T1	74	84	158		
T2	29	64	93		
T3	17	48	65		
T4	94	289	383		
Lymph node metastasis				11.876	**0.001**
No	102	164	266		
Yes	113	322	435		
TNM^b^ stage				25.218	**<0.001**
I	86	106	192		
II	38	105	143		
III	85	258	343		
IV	5	16	21		

^a^Values in bold are statistically significant.

^b^According to AJCC/UICC Classification for Carcinoma of the Stomach (Eighth edition).

### SNRPB enhances the proliferation and metastasis of GC both in vitro and in vivo

**Fig. 3:**
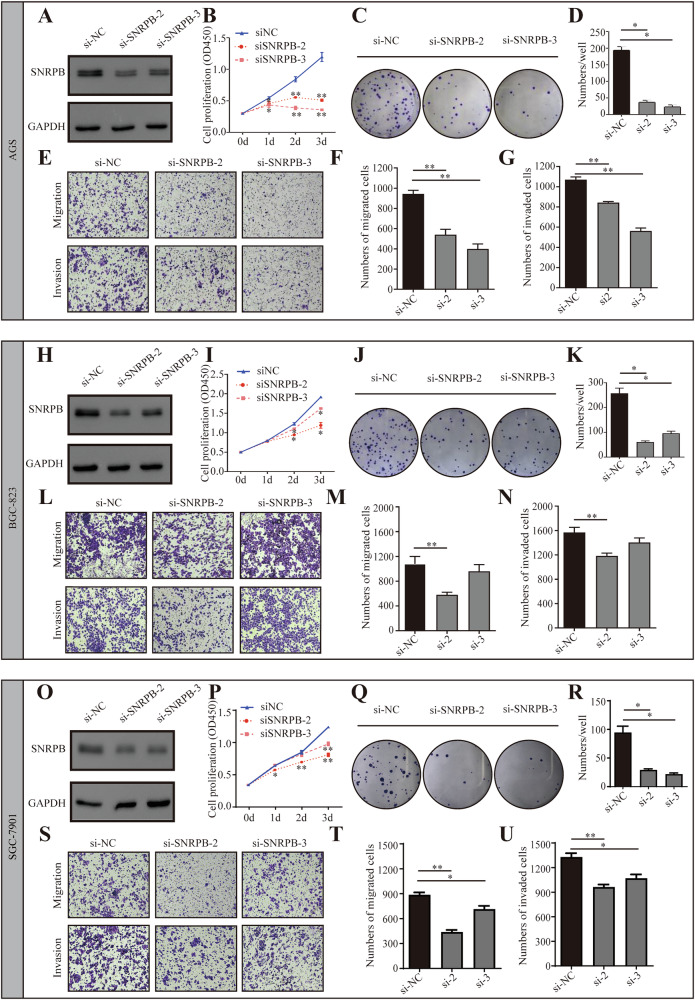
Knockdown of SNRPB inhibits the proliferation, migration, and invasion of gastric cancer cells. The western blot experiment verified the expression level of SNRPB in AGS (**A**), BGC (**H**), and SGC (**O**) cells after siSNRPB knockdown. The CCK-8 experiment demonstrated the proliferation ability of AGS (**B**), BGC (**I**), and SGC (**P**) cells after SNRPB knockdown. The clone formation experiment demonstrated that the proliferation ability of AGS (**C**, **D**), BGC (**J**, **K**), and SGC (**Q**, **R**) cells was reduced after SNRPB knockdown. The Transwell experiment showed that the migration and invasion abilities of AGS (**E**–**G**), BGC (**L**–**N**), and SGC (**S**–**U**) cells were reduced after SNRPB knockdown. *P* < 0.05*, *P* < 0.01**, *P* < 0.001***.

To assess the potential role of SNRPB in promoting the proliferation and metastasis of GC cells, two distinct small interfering RNAs (siRNAs) were designed to target and suppress SNRPB expression. The results demonstrated a significant reduction in the growth of AGS (Fig. 3A–D), BGC-823 (Fig. 3H–K), and SGC-7901 (Fig. 3O–R) cells upon SNRPB knockdown, as evidenced by decreased cell growth and impaired colony formation. Furthermore, the inhibition of SNRPB expression was found to impede the cell motility and invasive capabilities in these cell lines, as indicated by cell migration and invasion assays (Fig. 3E–G, L–N, S–U).

**Fig. 4:**
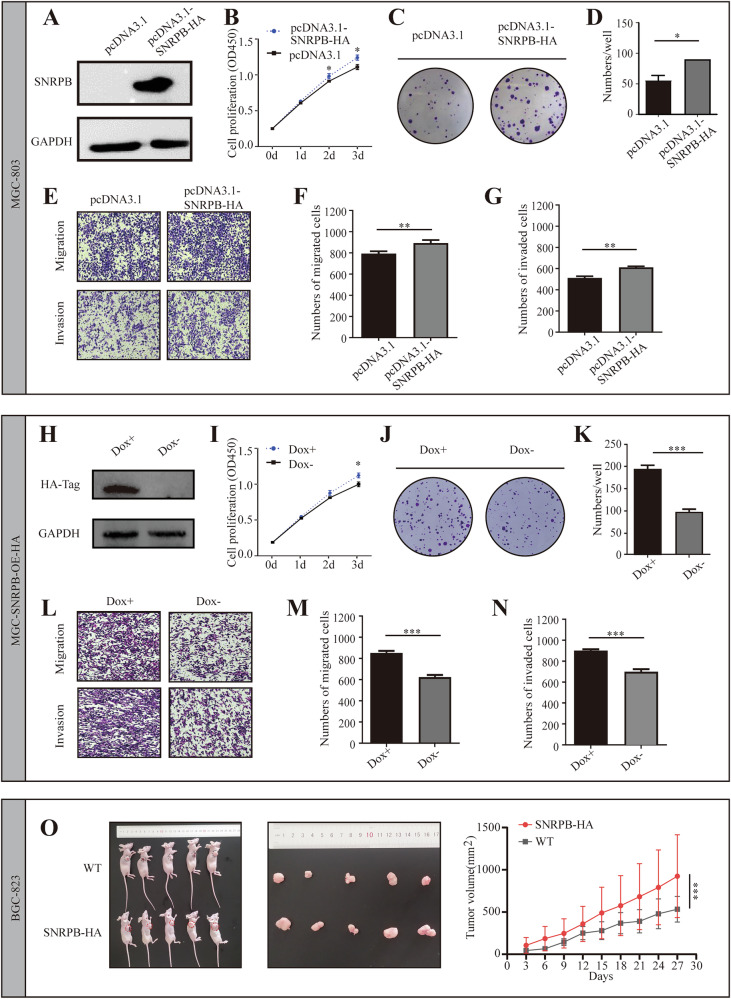
Overexpression of SNRPB can promote the proliferation, migration, and invasion of gastric cancer cells. **A**–**G** depict experiments utilizing recombinant plasmids to overexpress SNRPB in gastric cancer. **H**–**N** depict experiments where DOX was used to induce SNRPB-HA expression in MGC-SNRPB-HA cells. Western blot experiments showed the expression level of SNRPB in MGC cells transfected with the recombinant plasmid (**A**) and in MGC-SNRPB-HA cells induced by DOX (**H**). CCK-8 experiments (**B**, **I**) and clone formation assays (**C**, **D**, **J**, **K**) demonstrated that overexpression of SNRPB stimulated cell proliferation. Transwell experiments (**E**–**G**, **L**–**N**) indicated that overexpression of SNRPB increased cells’ migration and invasion capabilities. **O** In vivo experiments involving the subcutaneous injection of BGC-SNRPB-HA cells into nude mice confirmed that high expression of SNRPB promoted tumor growth. *P* < 0.05*, *P* < 0.01**, *P* < 0.001***.

To further confirm the importance of SNRPB in GC cell growth, an overexpression plasmid (pcDNA3.1-SNRPB-HA) was constructed and stably introduced into MGC-803 cells to generate MGC-SNRPB-HA cells. The ectopic expression of SNRPB in MGC-803 and MGC-SNRPB-HA cells notably enhanced cell proliferation and increased the migratory and invasive potential of these cells (Fig. 4A–N). Subsequent in vivo experiments involving the subcutaneous injection of BGC-SNRPB-HA cells into nude mice further supported the pivotal role of SNRPB in promoting tumor growth, as evidenced by the accelerated tumor growth rate, increased tumor size, and augmented tumor weight (Fig. 4O). These findings underscore the crucial role of SNRPB in regulating the proliferation and metastasis of GC cells.

### SNRPB is a key splicing factor and mediates the occurrence of abnormal AS events in downstream genes

**Fig. 5:**
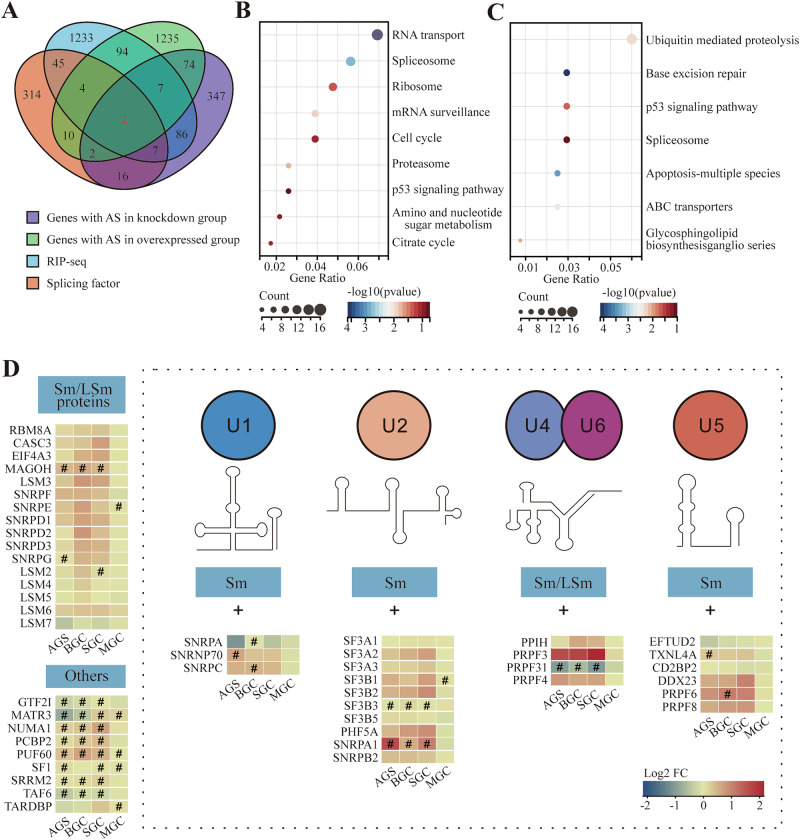
The effects of abnormal expression of SNRPB on genes causing AS events. **A** Venn diagram illustrating splicing factors that underwent alternative splicing events following abnormal expression of SNRPB. **B** Analysis of genes that underwent alternative splicing events in transcriptome sequencing after SNRPB knockdown using the KEGG database. **C** Analysis of genes that underwent alternative splicing events in transcriptome sequencing after SNRPB overexpression using the KEGG database. **D** Spliceosomal proteins and splicing factors underwent differential expression and alternative splicing events after abnormal expression of SNRPB. #: |Log_2_FC|>1 and *P* < 0.05.

We defined the experimental groups as the knockdown group (AGS, BGC-823, and SGC-7901 cells with siRNA interference targeting SNRPB expression) and the overexpression group (MGC cells overexpressing SNRPB with pcDNA3.1-HA-SNRPB). Transcriptome sequencing (RNA-seq) results showed that 490 genes in the knockdown group and 1428 genes in the overexpression group exhibited abnormal AS events. There were 85 genes in both groups, of which four were splicing factors (Fig. 5A). We conducted Kyoto Encyclopedia of Genes and Genomes (KEGG) pathway analysis on genes exhibiting abnormal AS events in both the knockdown and overexpression groups (Fig. 5B, C), and identified the spliceosome and p53 signaling pathways as two enriched pathways.

Abnormal SNRPB expression affected the differential expression and splicing of splicosome-related genes (Fig. 5D). Co-immunoprecipitation (Co-IP) was used to analyze the influence of abnormal SNRPB expression on the spliceosome. Following the knockdown and overexpression of SNRPB, we observed that exogenous SNRPB expression in GC cells led to direct or indirect binding to endogenous SNRPD1, SNRPD3, and SNRPE; however, it did not influence the endogenous expression of these proteins (Fig. S3A). Conversely, when endogenous SNRPB expression decreased, although the expression levels of these proteins were not significantly affected, the ability of SNRPB to directly or indirectly bind to SNRPE was reduced (Fig. S3B). This result indicates that the abnormal expression of SNRPB may have a specific impact on the formation of the SNRP protein complex.

### AS of the splicing factor PUF60 is regulated by SNRPB

**Fig. 6:**
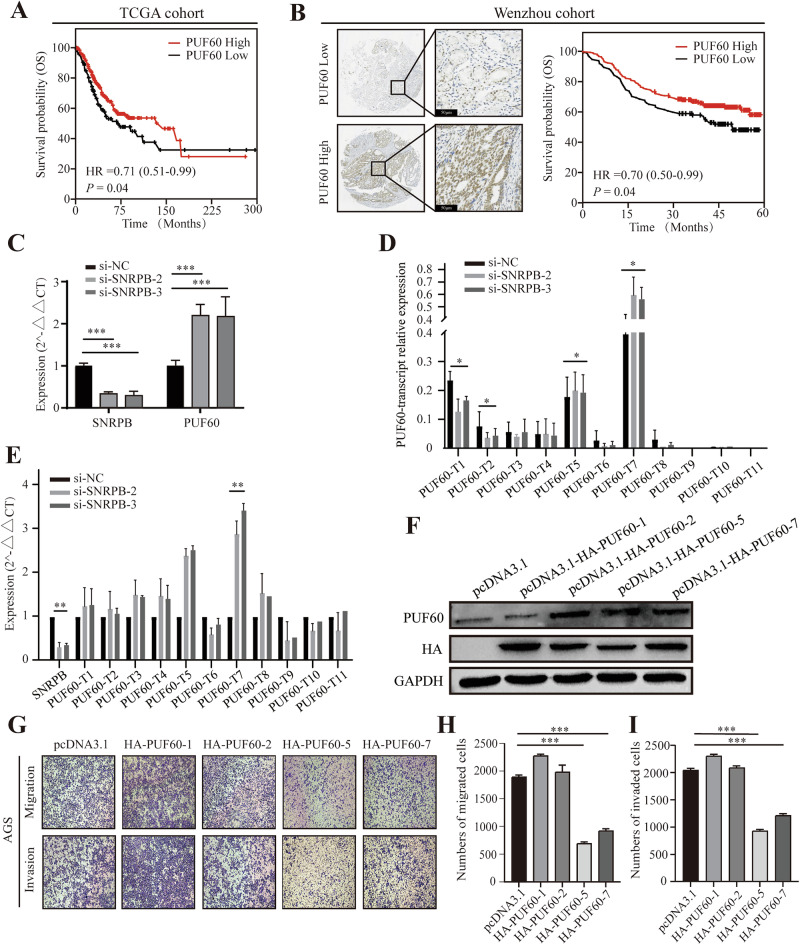
The alternative splicing of splicing factor PUF60 is regulated by SNRPB. **A** K–M survival curve of gastric cancer patients with high and low expression of PUF60 in the TCGA database. **B** The left figure shows representative IHC images of PUF60 staining for PUF60 low and PUF60 high groups, along with bar graphs at ×40 and ×400 magnification. The figure on the right illustrates the clinical significance of PUF60 low and PUF60 high groups in overall survival as assessed by Kaplan–Meier survival analysis. **C** qPCR detected the total mRNA expression of PUF60 by knocking down SNRPB. **D** qPCR detected the ratio of mRNA of 11 transcripts of PUF60 to total mRNA by knocking down SNRPB. **E** qPCR detected the mRNA expression levels of 11 transcripts of PUF60 by knocking down SNRPB. **F** Western blot analysis confirmed the successful transient transfection of PUF60 dominant transcript plasmids (pcDNA3.1-HA-PUF60-1, pcDNA3.1-HA-PUF60-2, pcDNA3.1-HA-PUF60-5, pcDNA3.1-HA-PUF60-7) in AGS cells. **G**–**I** The Transwell assay was performed to assess the migration and invasion abilities of cells following the transient transfection of PUF60 dominant transcript plasmids in AGS cells. *P* < 0.05*, *P* < 0.05**, *P* < 0.001***.

To further explore the association between SNRPB and spliceosome-related genes or factors, RNA immunoprecipitation sequencing (RIP-seq) was performed. SNRPB could directly act on 1489 mRNAs, including 58 splicing factors. After intersecting the splicing factors identified in the results of RIP-seq and transcriptome sequencing (SNRPB overexpression and knockdown groups), we discovered that only the mRNAs of two splicing factors (PUF60 and MATR3) directly bound to SNRPB (Fig. 5A, D). Notably, the previous results suggested that PUF60 is a differentially expressed splicing in GC tissues (Fig. 2B). IHC verified that PUF60 was highly expressed in GC tissues and was associated with a favorable prognosis (Fig. 6B), which is consistent with the TCGA dataset (Fig. 6A).

Our results demonstrated an upregulation in the overall mRNA expression level of PUF60 upon SNRPB knockdown (Fig. 6C), indicating that SNRPB primarily regulates PUF60 at the RNA level. We observed an upregulation of the mRNA expression levels of all 11 PUF60 transcripts when SNRPB was downregulated, with varying degrees of upregulation among the transcripts. Notably, transcripts 5 and 7 exhibited the most significant upregulation (Fig. 6D, E). The analysis of the transcript sequences revealed that AS events of PUF60 by SNRPB were primarily associated with the deletion of exon E5 (Fig. S4A). The results of the transwell assay indicated that PUF60 transcripts 1, 2, and 5, 7 had opposite biological functions: PUF60 transcripts 1 and 2 promoted the migration and invasion of GC cells, whereas 5 and 7 inhibited the migration and invasion of GC cells (Fig. 6F–I).

### SNRPB mediates the TP53 signaling pathway by regulating the AS of PUF60

**Fig. 7:**
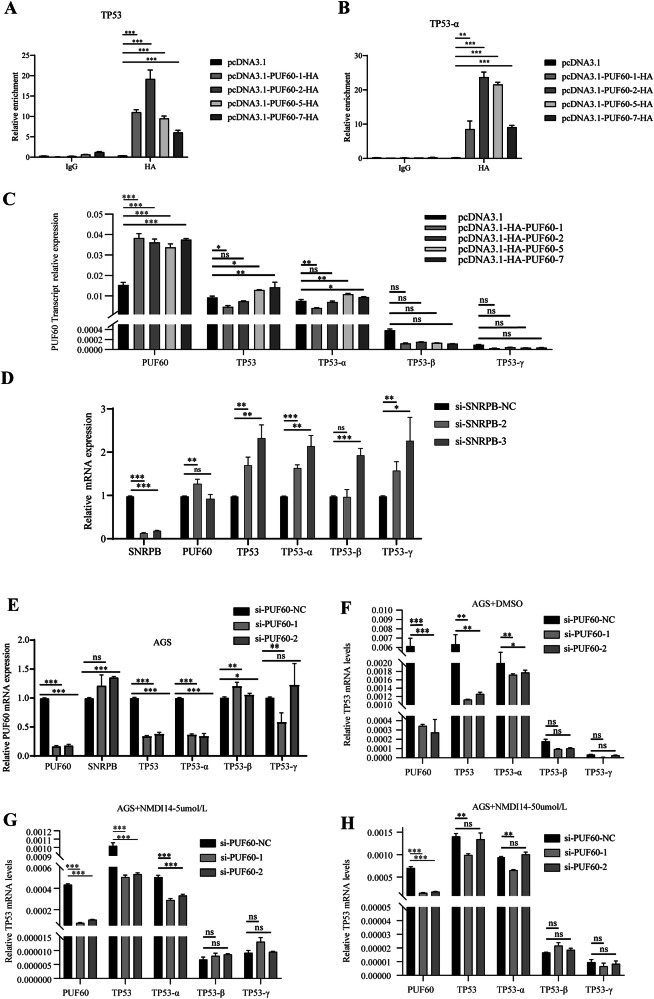
SNRPB mediates TP53 signaling pathway by regulating PUF60 alternative splicing. **A**, **B** The interaction between PUF60 transcripts and TP53 mRNA (**A**)/TP53-α mRNA (**B**) in AGS cells that overexpress PUF60 transcripts 1, 2, 5, and 7. **C** The effect of overexpression of PUF60 transcripts 1, 2, 5, and 7 on the RNA levels of each transcript of the TP53 gene. **D** The effect of SNRPB knockdown on the RNA level of each transcript of the TP53 gene. **E** The effect of SNRPB knockdown on the RNA level of each transcript of the TP53 gene. **F**–**H** The effects of adding DMSO (**F**), NMDI14 at 5 μmol/L (**G**), and NMDI14 at 50 μmol/L (**H**) on the RNA expression levels of each transcript of the TP53 gene based on knockdown of PUF60. *P* < 0.05*, *P* < 0.01**, *P* < 0.001***.

RNA-seq analysis revealed that the knockdown of PUF60 caused several p53 pathway-related genes to undergo AS (Fig. S5A). Subsequent RIP-seq experiments showed that PUF60 directly interacts with TP53, a key gene in the p53 pathway, and regulates AS (Fig. S5B). The TP53 gene produces three primary transcripts, p53α, p53β, and p53γ, with p53α being a well-known tumor suppressor (Fig. S4B). We further investigated the effects of various PUF60 transcripts on TP53. First, RIP experiments demonstrated that the proteins translated by PUF60 transcripts 1, 2, 5, and 7 interacted with both TP53 mRNA and TP53-α mRNA (Fig. 7A, B). Additionally, Moreover, qPCR revealed that the overexpression of PUF60 transcript 1 led to a reduction in the expression levels of both TP53 and TP53-α mRNA. This finding contrasted with the results observed following the overexpression of PUF60 transcripts 5 and 7 (Fig. 7C). Furthermore, when SNRPB is knocked down, the mRNA levels of both TP53 and TP53-α increase (Fig. 7D). Conversely, the mRNA levels of TP53 and TP53-α decreased upon PUF60 knockout (Fig. 7E).

NMDI14 is a well-known inhibitor of the nonsense-mediated mRNA decay (NMD) pathway. Upon knocking down PUF60, we treated the cells with dimethyl sulfoxide (DMSO) (Fig. 7F), NMDI14 at 5 μmol/L (Fig. 7G), and NMDI14 at 50 μmol/L (Fig. 7H) to assess the overall expression levels of TP53 and individual transcripts in GC cells. The results showed that, compared with the control group (DMSO group), the addition of NMDI14 reduced the impact of PUF60 knockdown on the overall mRNA expression levels of TP53 and its individual transcripts. Moreover, the higher the concentration, the more pronounced the effect.

## Discussion

AS is a common biological process in eukaryotes characterized by changes in the levels of splicing factors and dysregulation of downstream splicing targets, which are commonly observed in various types of cancers [24]. In 2018, an analysis of AS in 32 cancer types in 8705 patients found that tumors had 30% more AS events than normal samples, indicating the prevalence of abnormal AS events in cancer [25]. Researchers have demonstrated the general characteristics of abnormal AS events in various cancers, including colon [26], prostate [27], and liver cancer [28], using similar methodologies. Jun et al. employed variations in abnormal AS landscapes to classify subtypes of GC [8]. However, few researchers have recognized that systematic research on AS is crucial, particularly on the spliceosomes and splicing factors that play essential roles in the splicing process [29]. This study investigated the comprehensive landscape of aberrant AS events in GC and validated the findings using public databases. This analysis not only illustrated the landscape of abnormal variable splicing events in GC but also highlighted the commonalities among them. Importantly, we further examined the abnormal splicing events of splicing factors in GC and progressively explored closely related biomarkers and their mechanisms of action.

This study analyzed data from multiple databases and identified SNRPB as a key gene among 378 splicing factors that underwent abnormal AS events, depicting the AS spectrum of GC. SNRPB is an essential component of the U2 spliceosome; together with SNRPD3, SNRPD2, SNRPD1, SNRPE, SNRPF, and SNRPG, they form a circular complex. SNRPB is a crucial common element among five snRNPs (U1, U2, U4, U5, and U6) [30]. The dysregulation of SNRP impairs pre-mRNA splicing, leading to the production of unexpected mRNA variants from a single gene. These variants translate into new proteins that may play a significant role in tumors [31–33]. Recent studies have shown that SNRPB is highly expressed in various tumor tissues and plays a role in regulating tumor progression [14, 15, 34, 35]. This study used a combination of bioinformatics and in vivo and in vitro experiments to systematically investigate the mechanism and clinical prognostic value of SNRPB in the development of GC from the perspective of AS dysregulation. In this study, we found that endogenous SNRPB is abnormally overexpressed in GC tissues and various GC cell lines. This overexpression was associated with poor patient prognosis. Simultaneously, overexpression of SNRPB promoted the proliferation, migration, and invasion of GC cells and facilitated the growth of xenograft tumors in vivo. These results indicate that SNRPB plays a role in promoting malignant phenotypes during the progression of GC. These results are consistent with those of previous studies on other malignant tumors and further demonstrate that SNRPB has the potential to serve as a biomarker for GC. In addition, researchers, such as Zhi Zeng [36], have utilized whole-genome CRISPR-Cas9 technology to demonstrate that SNRPB is an essential gene in GC AGS cells, with significant decreases in cell survival and proliferation rates following knockout.

Previously, Saltzman et al. found that the knockdown of SNRPB in HeLa cells affected AS events in genes involved in RNA processing, such as spliceosome components and other splicing factors [37]. Through transcriptome sequencing, we discovered that the aberrant expression of SNRPB not only affects the expression levels of certain genes but also induces abnormal AS events in some genes, including PUF60. PUF60 is a 60 kDa protein that participates in pre-mRNA splicing and regulates Myc gene expression [38]. Previous studies have also found that abnormal PUF60 expression promotes the progression of various malignant tumors [19, 39, 40]. Furthermore, high expression of PUF60 in various cancers indicates poor prognosis [19, 20, 41, 42]. However, the results of this study suggest that high PUF60 expression in GC indicates a better prognosis. There may be bias caused by the small number of samples used for IHC. In addition, it is important to verify the direct interaction between the SNRPB protein and PUF60 RNA using RIP-seq. We found that SNRPB knockdown induced exon-skipping events in PUF60 mRNA through AS, resulting in an increased proportion of the two dominant transcripts of PUF60. This, in turn, inhibits GC metastasis. These phenomena indicate that abnormal SNRPB expression can regulate the occurrence of abnormal AS events in PUF60 cells and promote the progression of GC.

**Fig. 8:**
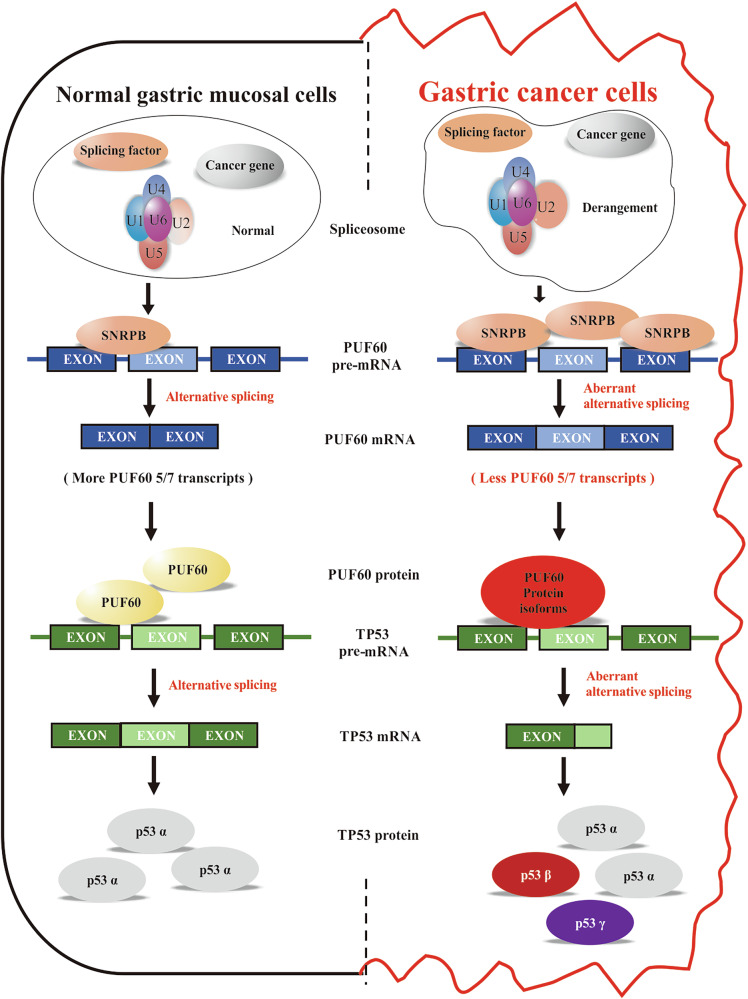
The mechanism diagram of SNRPB regulating abnormal splicing of PUF60 and indirectly affecting TP53 signaling pathway.

In addition, we conducted transcriptome sequencing after the downregulation of PUF60 expression. We found that the abnormal expression of PUF60 promoted AS events in many genes related to the p53 pathway, including TP53. As a classic tumor regulatory pathway, the p53 pathway is mainly involved in regulating DNA repair and controlling the cell cycle, apoptosis, and differentiation [43]. The TP53 gene expresses multiple p53 protein isoforms through AS, with p53α being the most common isoform. This isoform inhibits cancer development by regulating the cell cycle, mediating DNA damage repair, and inducing apoptosis [44]. Altering the expression levels of SNRPB in GC cells induced aberrant AS events in genes associated with the p53 pathway. However, RIP-seq using specific antibodies against SNRPB proteins did not detect the enrichment of key genes in the p53 pathway. In contrast, RIP using specific antibodies against the PUF60 protein detected the interaction between the PUF60 protein and TP53 RNA. Additionally, the proteins translated from the major PUF60 transcripts 1, 2, 5, and 7 were also shown to interact with TP53 RNA or TP53-α RNA. Simultaneously, by overexpressing the predominant PUF60 transcripts in GC cells, we observed significant changes in the expression levels of TP53 and its three primary transcripts. Additionally, different PUF60 transcripts exhibit contrasting effects on the regulation of TP53. Therefore, we believe that SNRPB indirectly influences the expression level of TP53 by regulating the AS of PUF60, thereby exerting a cancer-promoting effect (Fig. 8).

Studies of aberrant AS events in GC have yielded valuable insights into the molecular mechanisms underlying tumorigenesis and progression. However, our study has some limitations, and several areas require further attention and refinement. First, the sample size was relatively small, which may have affected the generalizability of our findings. To address this limitation, we plan to expand our cohort in future studies to include more diverse and larger patient groups, thereby enhancing the statistical power and external validity of our results. Second, although our in vitro and in vivo experiments demonstrated the role of SNRPB in GC cells, the clinical relevance and therapeutic potential of our findings must be validated in larger clinical trials. We collaborated with clinical partners to conduct prospective studies to evaluate the prognostic significance of the SNRPB and its spliceosome-related genes in patients with GC. Third, our study focused on the impact of SNRPB on the spliceosome and TP53 signaling pathways. However, the intricate regulatory network of AS is likely to involve additional pathways and factors. Future research should investigate the interaction of SNRPB with other signaling pathways and the broader splicing landscape in GC.

## Conclusion

In summary, this study is the first to confirm, at multiple levels, that SNRPB is overexpressed in GC and that this overexpression is associated with poor prognosis. Our findings provide evidence that SNRPB interacts with PUF60 to regulate AS events and their effect on TP53 expression in GC. We hope that the results of this study contribute to a more comprehensive understanding of AS events in GC. Furthermore, we aimed to build on our current findings to explore new therapeutic targets and strategies for treating GC.

## Materials and methods

### Bioinformatics analysis

The seven types of ASE data: alternate acceptor (AA); alternate donor (AD); alternate promoter (AP); alternate terminator (AT); exon skip (ES); mutually exclusive exons (ME); retained intron (RI), along with the Present Spliced In (PSI) values, were available in the TCGASpliceSeq database. Alternative 3’SS (A3SS), alternative 5’SS (A5SS), skipped exon (SE), retained intron (RI), and mutually exclusive exons (MXE) AS events of GEO datasets (GSE172032) and 3 self-tested GC and paired paracancerous tissue data were evaluated using rMATS (v4.1.2). Then, functional enrichment analysis (Gene Ontology Molecular Function, Biological Process, Cellular Component pathways) was performed on each gene dataset using the GOStats R package. UpSet was applied to visualize the associations between splicing factors and each type of AS event.

GEO2R (http://www.ncbi.nlm.nih.gov/geo/geo2r/) was used to perform gene differential expression analysis to find differentially expressed genes (DEGs) between normal and tumorous tissues across five GEO datasets (GSE13911, GSE29272, GSE30727, GSE51575, GSE56807). Next, the Kaplan–Meier plotter (https://kmplot.com/analysis/) and R2 database (http://r2.amc.nl) were employed to examine the association between five gene expression levels and the prognosis of GC patients.

### Clinical specimens

We obtained 17 frozen GC tissues and paired tumor-adjacent normal gastric tissues for total RNA extraction and qRT-PCR). In addition, we collected the clinicopathological characteristics and paraffin-embedded GC tissues from GC patients who underwent surgical treatment between 2006 and 2018, totaling 701 cases. Among them, 69 GC patients also had paraffin-embedded paracancerous tissue (at least 10 cm away from the negative edge). We also collected paraffin-embedded normal gastric mucosa tissues from 23 bariatric patients. These tissues were used for the IHC of SNRPB. The inclusion criteria for GC patients are as follows: (1) all patients’ tissue specimens must have received a pathological diagnosis of GC from at least two professional pathologists; (2) patients must not have undergone radiotherapy or chemotherapy prior to surgery; (3) they must not have any other concurrent malignant tumors; (4) each patient must have been followed up to gather pathological reports and monitor disease progression, with no patients lost to follow-up. According to the American Joint Committee on Cancer Staging, the patient’s demographic and clinicopathological characteristics include gender, age, serum CEA, serum CA199, Lauren classification, tumor diameter, differentiation level, depth of invasion, lymph node metastasis, and TNM stage. This study was approved by the Ethics Review Board of the First Affiliated Hospital of Wenzhou Medical University, and written informed consent was obtained from all the study participants included.

### Immunohistochemistry

Paraffin-embedded tissue samples were cut into 5-μm-thick sections. The tissue sections were deparaffinized using xylene and then rehydrated with a series of graded ethanol solutions. Dewaxed tissue sections were boiled in 10 mM citrate buffer (pH 6.0) for 100 min for antigen retrieval. Completely cover the tissue with a 3% H_2_O_2_ solution and incubate it at room temperature for 10 min to inhibit endogenous peroxidase activity. The tissue sections were then incubated with goat serum (Solarbio, Beijing, China) for 30 min at room temperature, followed by incubation with antibodies for 3 hours at room temperature. After rinsing three times with PBST, visualization was performed using a Dako EnVision FLEX detection system (Dako, Carpinteria, CA, USA) following the manufacturer’s instructions. The sections were counterstained with hematoxylin, dehydrated, and then sealed with neutral gum. The results were interpreted and scored by two professional pathologists. Scoring criteria: The staining intensity was scored on a scale of 0 (negative), 1 (weak), 2 (moderate), and 3 (strong). The scoring of the positive expression was divided into 0 (0%), 1 (<25%), 2 (25–50%), 3 (50–75%), and 4 (>75%), based on the percentage of positively stained cells. The final score for each specimen was determined by multiplying the two scores together. We define values of six or less as indicating low expression and values greater than six as indicating high expression.

### Cell culture

AGS, BGC-823, SGC-7901, and MGC-803 GC cell lines were purchased from the Cell Bank of the Academy of Sciences in Shanghai, China. These cell lines were maintained in Roswell Park Memorial Institute – 1640 (RPMI-1640) or Dulbecco’s modified Eagle’s medium (DMEM; Gibco) complete medium containing 10% fetal bovine serum (FBS; Gibco) and incubated at 37 °C with 5% CO_2_.

### Transfection

The open reading frame fragment of SNRPB, tagged with HA, was inserted into the pcDNA3.1(+) vector between the KpnI and NotI restriction sites. The constructed plasmid (pcDNA3.1(+)-SNRPB-HA) was verified through sequencing. The HA tag can be used to confirm the expression of SNRPB-HA in GC cells. RiboBio (Guangzhou, China) designed and synthesized two siRNAs targeting SNRPB, with the following sequences: siSNRPB-2: 5’- CAAGCCAAAGAACTCCAAA -3’, siSNRPB-3: 5’- GGACCTCCTCCCAAAGATA -3’. Prepare the siRNA storage solution (20uM) according to the manufacturer’s instructions. Both the plasmids and siRNAs were used for cell transfection.

When the cell growth density in each well of the 6-well plate reached approximately 60-70%, siSNRPB and control siNC (or plasmid pcDNA3.1(+)-SNRPB-HA and control pcDNA3.1(+)) were used for transfection. Added siRNA to each well of the 6-well plate to achieve a final concentration of 50 nM (or added plasmid to achieve a final concentration of 1 μg/mL. The medium was changed after 6 hours, and the cells were used for subsequent experiments. All transfections were performed using Lipofectamine 2000 (Invitrogen) according to the manufacturer’s instructions.

### CCK-8

For the Cell Counting Kit-8 (CCK-8) assay, GC cells were seeded into 96-well plates at a density of 5000 cells per well. After 24, 48, and 72 h of transfection, 10 μl of CCK-8 reagent (Dojindo, Kumamoto, Japan) was added into each well and incubated for 2 h in a 37 °C, 5% CO_2_ cell culture incubator. The absorbance was measured at a wavelength of 450 nm. All experiments were carried out in triplicate.

### Cell colony formation assay

For the cell colony formation assay, GC cells were transfected for 48 h and then seeded in a 6-well plate at a density of 500 cells per well. The cells were cultured in a cell incubator at 37 °C and 5% CO_2_ for 2 weeks, with media replacement every 3 days with one culture medium. Then, cell colonies were fixed with 4% paraformaldehyde for 10 min and stained with a 0.5% crystal violet solution at 25 °C for 10 min. Colonies containing more than 20 cells were counted. All experiments were performed in triplicate.

### Transwell migration and invasion assays

For the cell migration and invasion assay, 1 × 10^5^ cells were suspended in 100 μl of serum-free medium and placed in the upper chamber of a Transwell chamber, with or without Matrigel (Corning, NY, USA). In the bottom chamber, 600 μl of serum-containing medium was added. The migrated or invaded cells were fixed at the bottom of the membrane using 4% paraformaldehyde and then stained with a 0.5% crystal violet solution. The number of cells in five randomly selected fields per well was counted under a microscope (Leica, London, UK, with a × 10 eyepiece and a × 40 objective lens) to determine the cell count in each group.

### Western blotting

Total proteins from cells were extracted with the RIPA lysis buffer (Beyotime) supplemented with cocktail (Sigma-Aldrich, St. Louis, MO, USA). The equal amounts of protein lysates were separated on a 10% SDS-PAGE gel and transferred to polyvinylidene fluoride (PVDF) membranes (Bio-Rad, Hercules, CA, USA). The membranes were then blocked with 5% skim milk at 25 °C for 1 h. The membranes were then incubated with specific antibodies overnight at 4 °C, followed by incubation with horseradish peroxidase-conjugated secondary antibodies for 2 h. The visualization was done using a Bio-Rad imaging system.

### RT-PCR

Total RNA was extracted from cells and GC tissues using TRIZOL Reagent, and then reverse transcribed into cDNA using the FSQ-301 reverse transcription kit (TOYOBO, Tokyo, Japan). PCR was performed according to the instructions of the 2×Phanta Evo HS Master Mix kit (Vazyme), with GAPDH as the control. Quantitative PCR was performed according to the instructions of the QuantiNova SYBR® Green kit (QIAGEN), using GAPDH as the internal reference. See the attachment for primer sequences.

### RNA-binding protein immunoprecipitation (RIP)

The RIP process was performed according to the instructions of the RNA-Binding Protein Immunoprecipitation Kit (Millipore). The target antibody and IgG antibody used to incubate the magnetic beads were added according to the maximum amount of 5 µg specified in the instructions. At least approximately 4 × 10^7^ cells were prepared, and the cell suspension was collected using an enzyme-free cell scraper. Utilized the cell lysis solution provided in the kit to lyse the cells. The lysate supernatant was incubated with magnetic beads that had been pre-coated with anti-SNRPB or anti-IgG overnight at 4 °C. The RNA bound to the magnetic beads was then extracted for sequencing analysis.

### In vivo analysis

Female BALB/C/nu nude mice, 4 weeks old and weighing 18–22 g, were purchased from the Animal Center of Hangzhou Medical College and housed in a specific pathogen-free animal laboratory. Feeding and management were carried out by professional laboratory personnel. All animals complied with the Wenzhou University Laboratory Animal Care and Use Policy.

The nude mice were randomly divided into two groups, each consisting of five mice. The BGC-SNRPB-OE-HA cell line was injected subcutaneously into the right back of the nude mice at a concentration of 1 × 10^7^ cells per mouse. Feed drinking water containing DOX (concentration) or control drinking water every day. Observe the condition of the nude mice and measure the length and width of the tumors. Change the water and measure it every 3 days. After being euthanized with isoflurane approximately four weeks later, the tumors were excised, categorized into groups, and their sizes were measured. The calculation formula for tumor volume is as follows: volume (*V*) = (long diameter × short diameter^2)/2.

### Statistical analysis

The differences between the components were assessed using chi-square tests and independent samples *t* tests. The prognostic significance of SNRPB was assessed using Kaplan–Meier plots. A Cox regression model was used to analyze the independent risk factors for survival. The statistical analysis and result visualization were performed using SPSS Statistics (version 23.0; IBM SPSS, Chicago, IL), GraphPad Prism 7 (GraphPad Software, CA, USA), and the R&R studio (R&R studio) software. Statistical significance was set at *P* < 0.05*, *P* < 0.01**, *P* < 0.001***.

## Supplementary information

aj-checklist

SupplementaryFigureS1-5

Western Blot original image

## Data Availability

All relevant data are fully presented in the figures and tables of this article. Additional data can be obtained by contacting the corresponding author.
