# Correction: SNRPB promotes gastric cancer progression by regulating aberrant splicing of PUF60

**DOI:** 10.1038/s41419-026-08783-1

**Published:** 2026-05-13

**Authors:** Dan Xiang, Jiaxin Yang, Miaofang Xiao, Cong Long, Yangxuan Lin, Chenchen Mao, Xin Liu, Dianfeng Mei, Wangkai Xie, Zheng Han, Chenbin Chen, Xiaoming Lin, Xian Shen, Xiangyang Xue, Tanzhou Chen

**Affiliations:** 1 Wenzhou Collaborative Innovation Center of Gastrointestinal Cancer in Basic Research and Precision Medicine, Wenzhou Key Laboratory of Cancer-related Pathogens and Immunity, Experiemtial Center of Basic Medicine, Department of Microbiology and Immunology, Institute of Molecular Virology and Immunology, School of Basic Medical Sciences, Wenzhou Medical University, Wenzhou, China; 2 Department of Gastrointestinal Surgery, Taizhou Hospital of Zhejiang Province affiliated to Wenzhou Medical University, Taizhou, China; 3 GMU-GIBH Joint School of Life Sciences, The Guangdong-Hong Kong-Macao Joint Laboratory for Cell Fate Regulation and Diseases, Guangzhou Medical University, Guangzhou City, China; 4 Hantai District Maternal and Child Health Hospital, Dongguan, China; 5 Department of Thoracic Surgery, The First Affiliated Hospital of Wenzhou Medical University, Wenzhou, China; 6 Department of General Surgery, The Second Affiliated Hospital and Yuying Children’s Hospital of Wenzhou Medical University, Wenzhou, China; 7 Department of Clinical Laboratory, Xuyong County People’s Hospital, Luzhou City, China; 8 Department of General Surgery, The First Affiliated Hospital of Wenzhou Medical University, Wenzhou, China; 9 The Department of Gastroenterology and Hepatology, The First Affiliated Hospital of Wenzhou Medical University, Wenzhou, P.R. China

**Keywords:** Gastric cancer, Tumour biomarkers

Correction to: *Cell Death & Disease* 10.1038/s41419-025-08011-2, published online 07 October 2025

There were some mistakes in Figure 6G-I:

1. Figure 6G,a figure layout mistake in the migration and invasion images
2. Figure 6H-I are the statistical graphs corresponding to Figure 6G, which have been re-statistically analyzed.

**Figure 6 Uncorrected** 
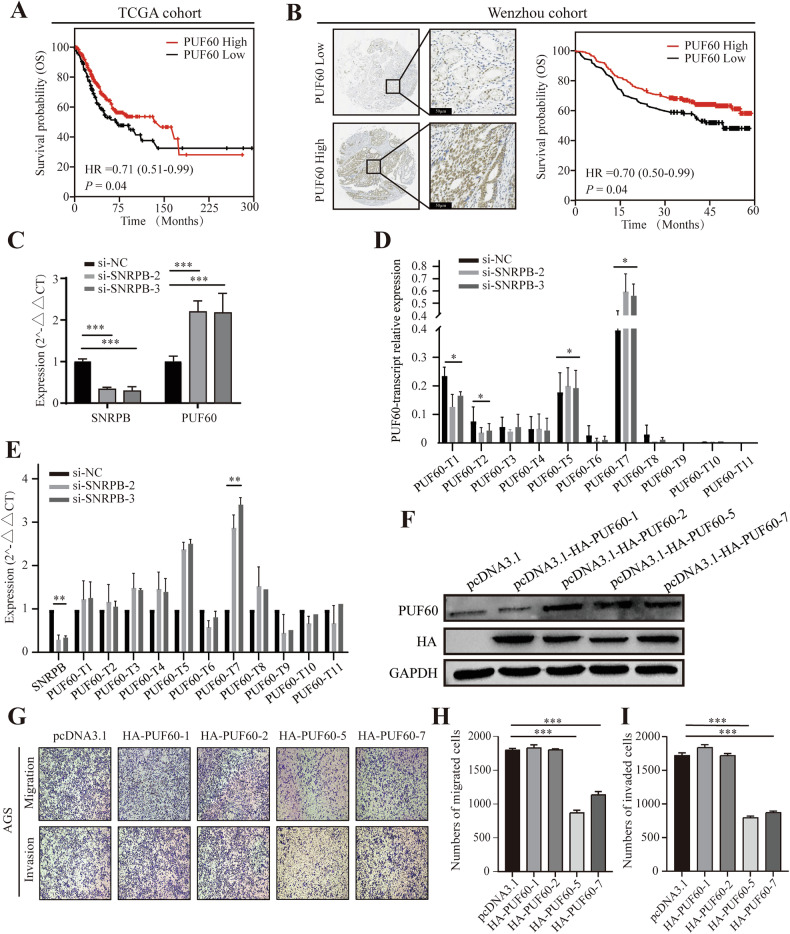


**Figure 6 Corrected** 
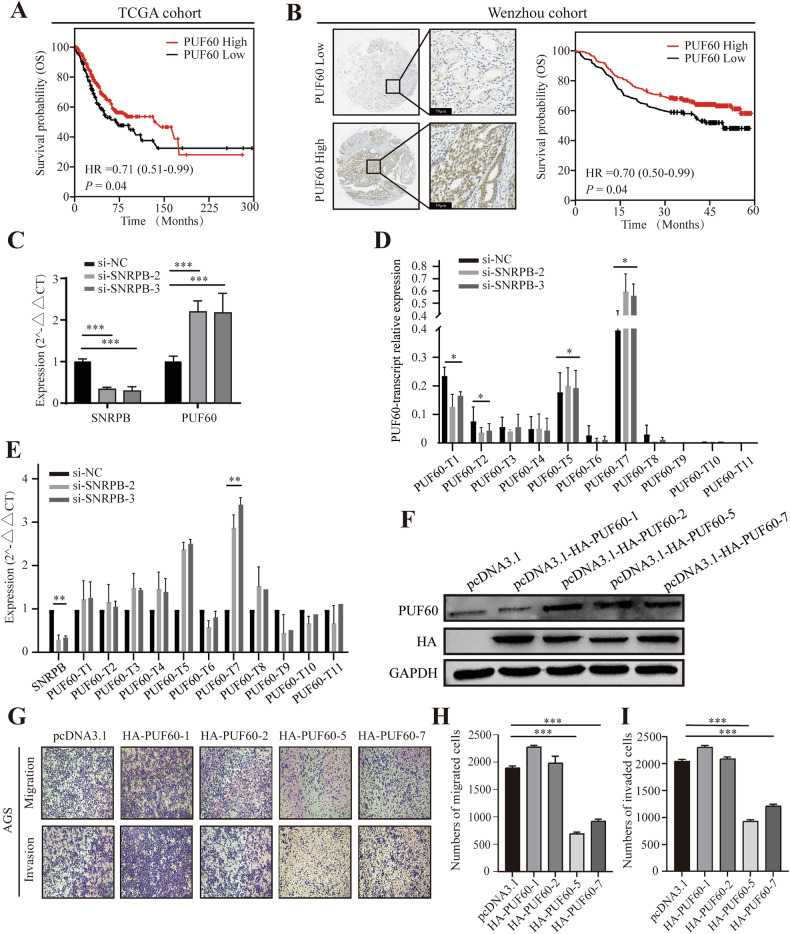


The original article has been corrected.

