# Supplementary material for: SNRPB promotes gastric cancer progression by regulating aberrant splicing of PUF60
Source: Cell Death Dis. 2025 Oct 7;16(1):709. doi: 10.1038/s41419-025-08011-2 (PMC12504745; doi:10.1038/s41419-025-08011-2)
Supplement: Supplementary file 2 — SupplementaryFigureS1-5 [file 41419_2025_8011_MOESM2_ESM.pdf]

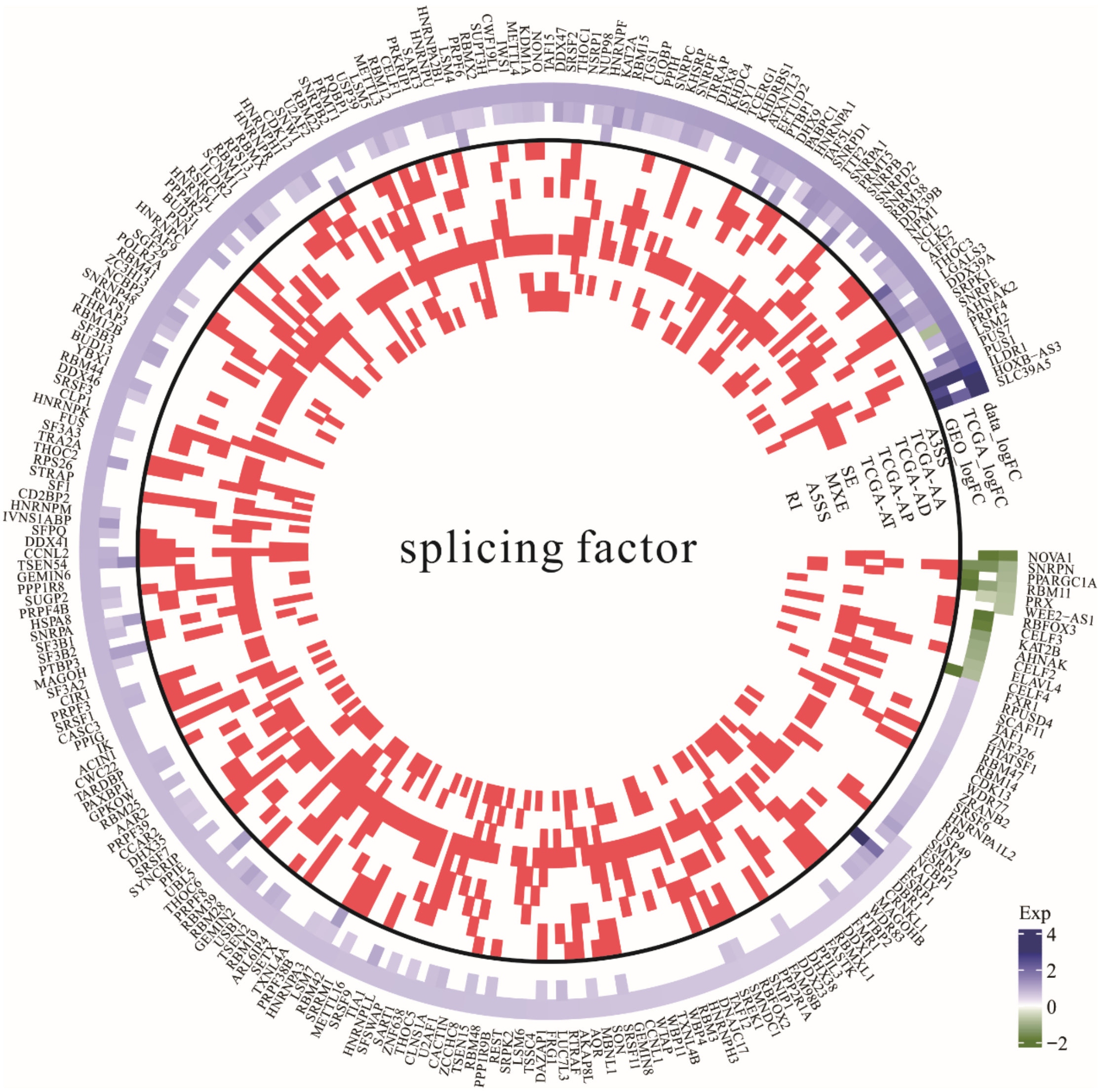

Figure S1. The circular heatmap illustrating the distribution and frequency of alternative splicing events among 260 splicing factors and their differential expression in the three datasets. The 260 splicing factors were identified by comparing the data from self-tested gastric cancer and adjacent cancer tissues with public databases (TCGA database, GEO database) and identifying the intersection. The inner circle represents the alternative splicing events that occurred. The outer circle represents the expression difference, and from the outside to the inside are GEO dataset (GSE172032), TCGA database, and the samples submitted for testing.

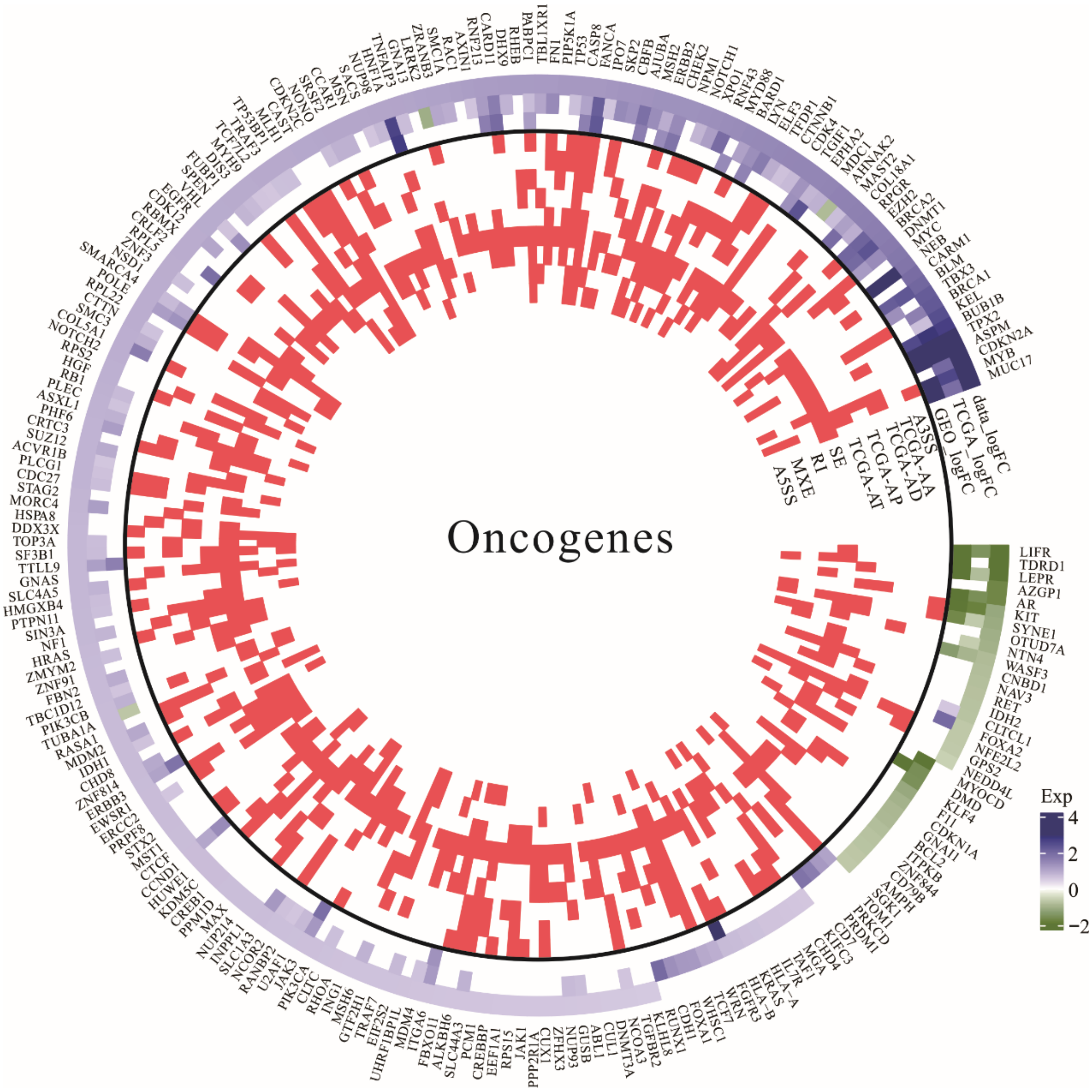

Figure S2. The circular heat map illustrating the distribution and frequency of alternative splicing events among 390 oncogenes and their differential expression in the three datasets. The 390 oncogenes were identified by comparing the data from self-tested gastric cancer and adjacent cancer tissues with public databases (TCGA database, GEO database) and identifying the intersection. The inner circle represents the alternative splicing events that occurred. The outer circle represents the expression difference, and from the outside to the inside are GEO dataset (GSE172032), TCGA database, and the samples submitted for testing.

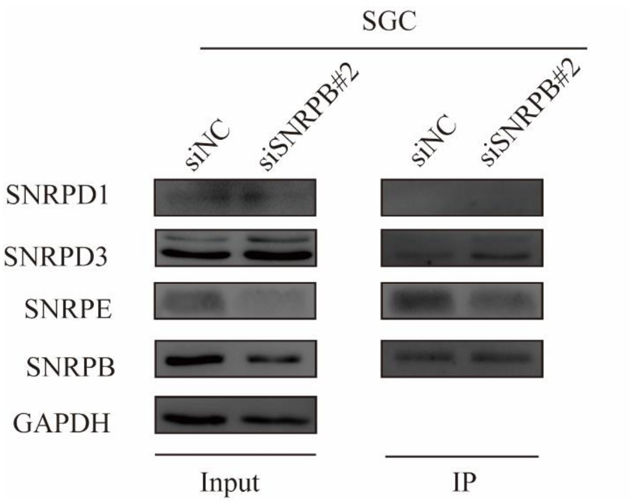

Figure S3. Co-immunoprecipitation (Co-IP) technology was used to analyze the effect of abnormal SNRPB expression on the SNRP protein complex. knockdown of SNRPB were performed to detect the expression levels of SNRPD1, SNRPD3, and SNRPE, as well as their binding with SNRPB.

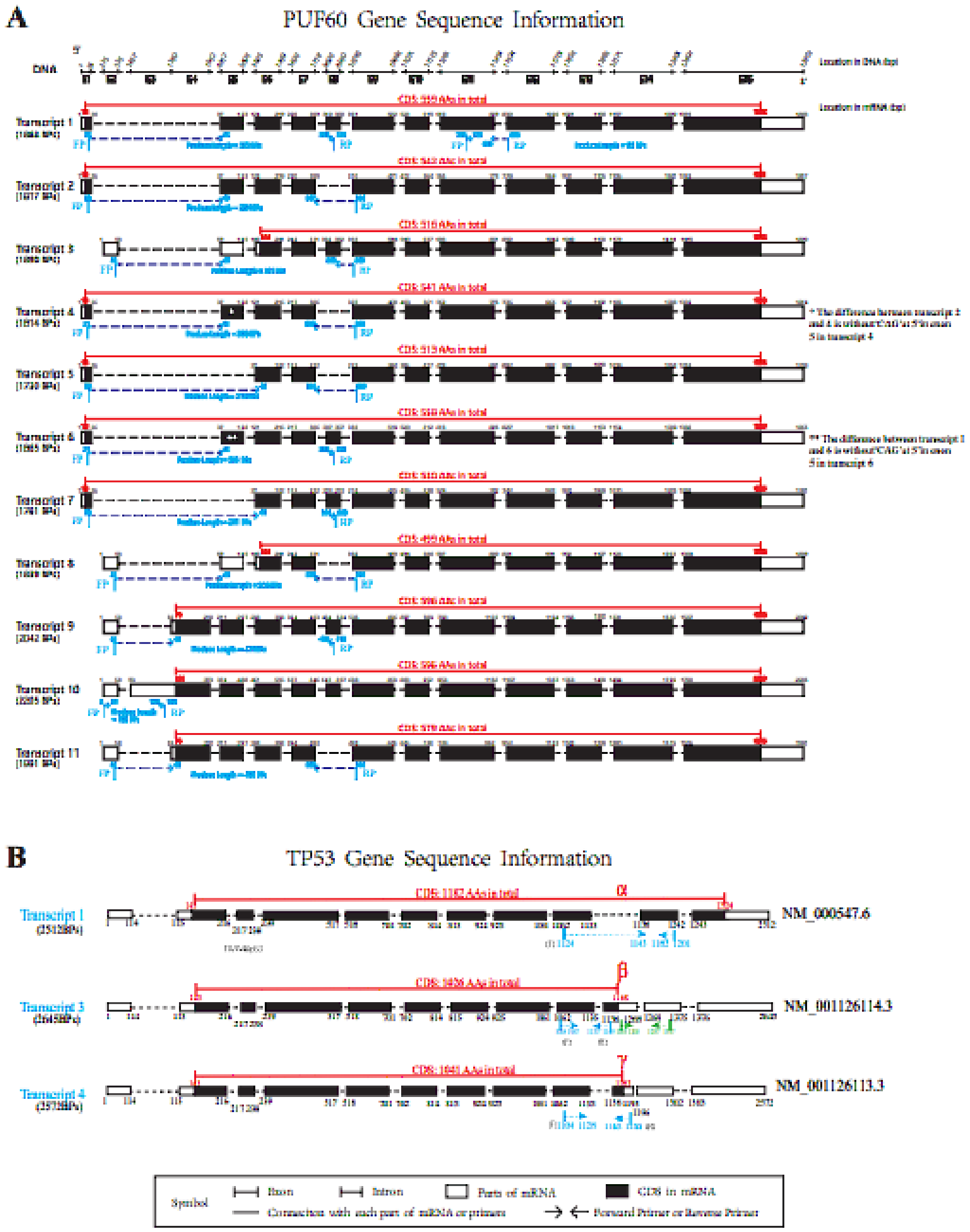

Figure S4. The schematic diagram of primer design. (A) Information on each transcript and specific primer for PUF60. (B) Information on each transcript and specific primer for TP53.

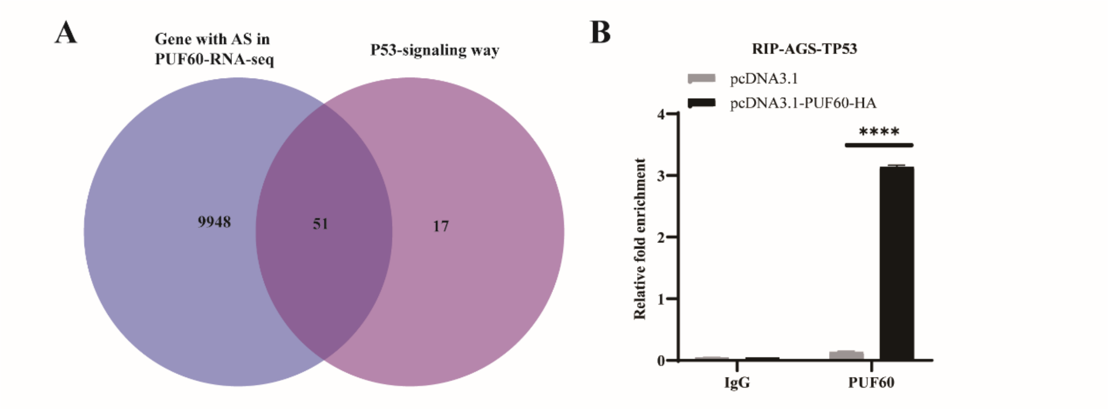

Figure S5. Bioinformatics analysis suggests that abnormal expression of PUF60 may cause abnormal alternative splicing events in genes involved in the p53 signaling pathway. (A) Overlapping analysis of genes identified by PUF60-RNA-seq and P53 signaling pathway. (B) The interaction between PUF60 and TP53 mRNA was analyzed in AGS cells overexpressing PUF60 by RIP assay.
