# Supplementary figures and images for: SNRPB promotes gastric cancer progression by regulating aberrant splicing of PUF60
Source: Cell Death Dis. 2025 Oct 7;16(1):709. doi: 10.1038/s41419-025-08011-2 (PMC12504745; doi:10.1038/s41419-025-08011-2)

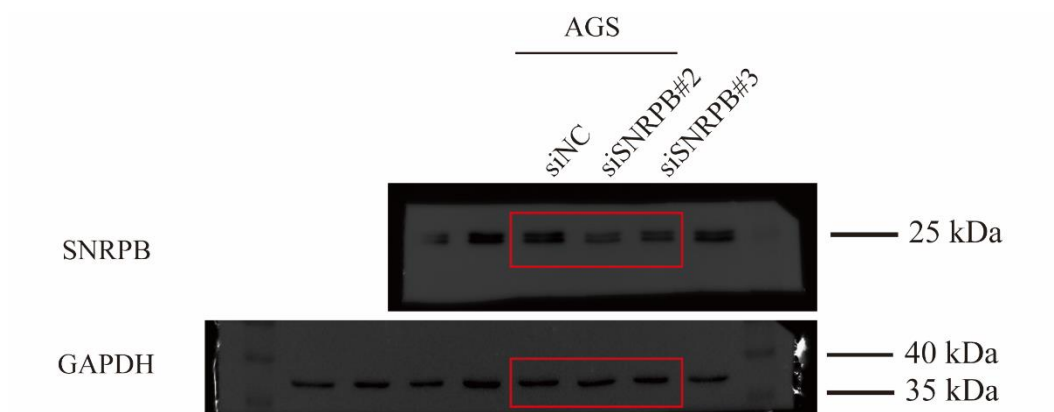

**Figure 3A**

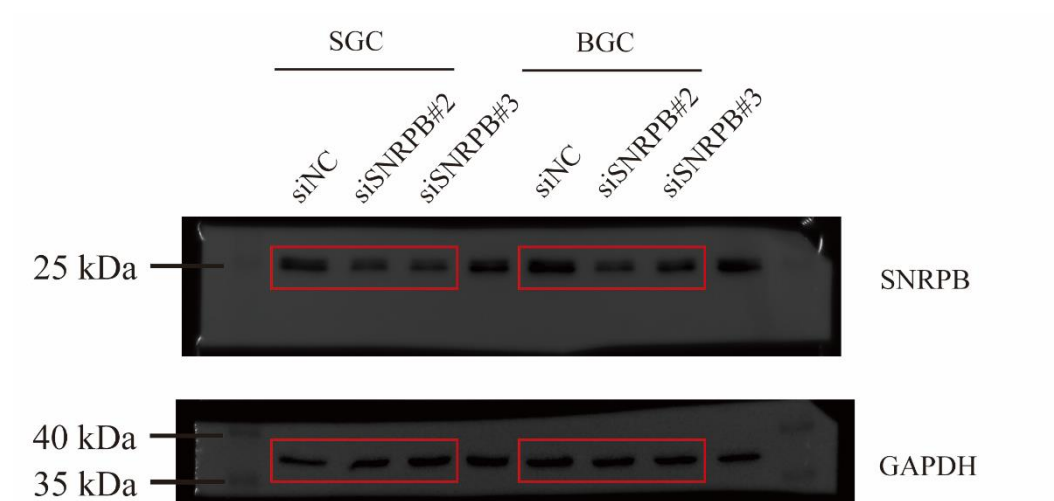

**Figure 3H and 3O**

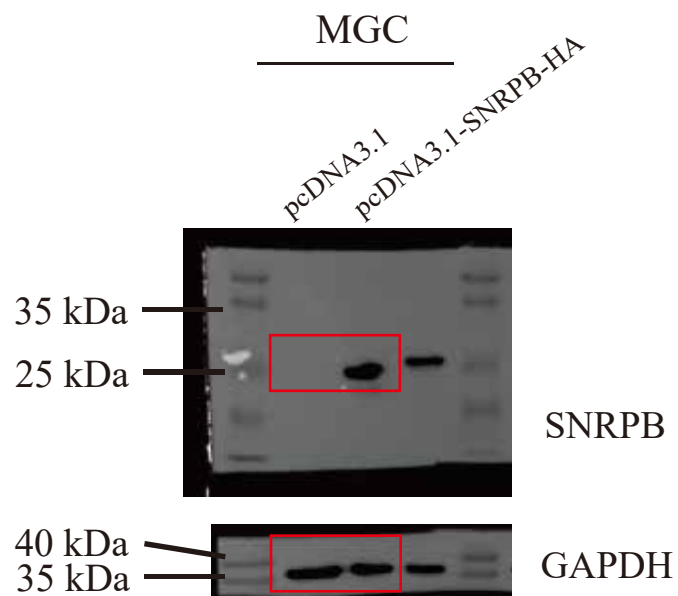

Figure 4A

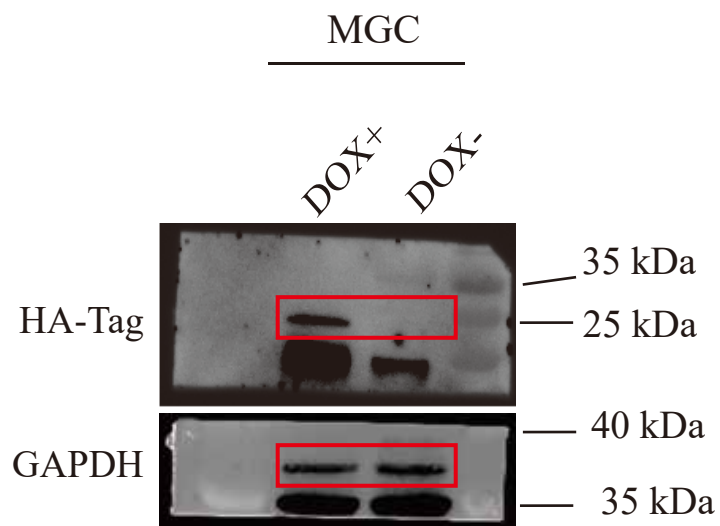

Figure 4H

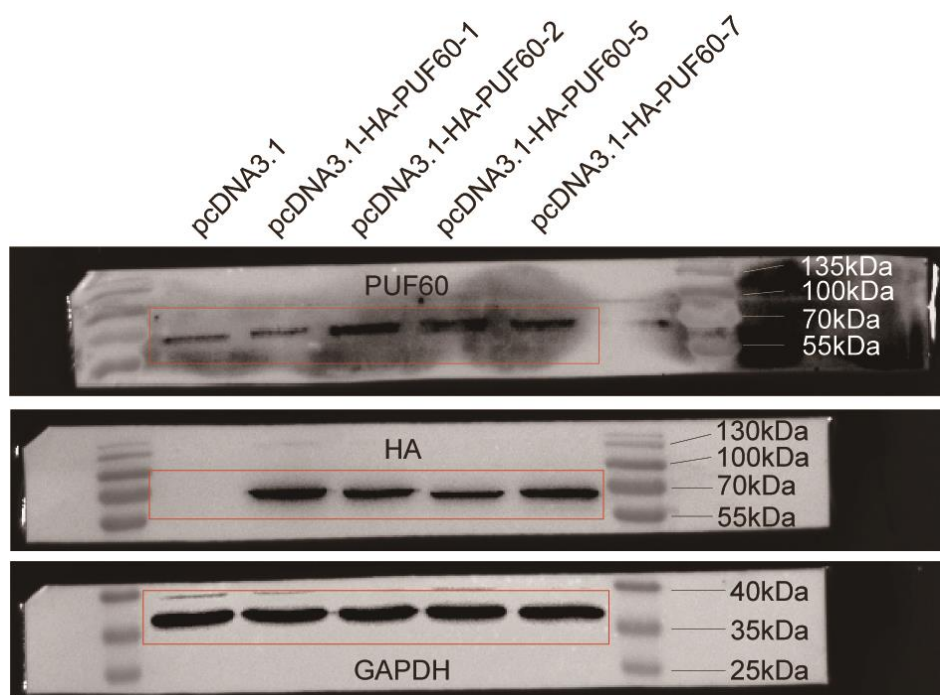

Figure 6F

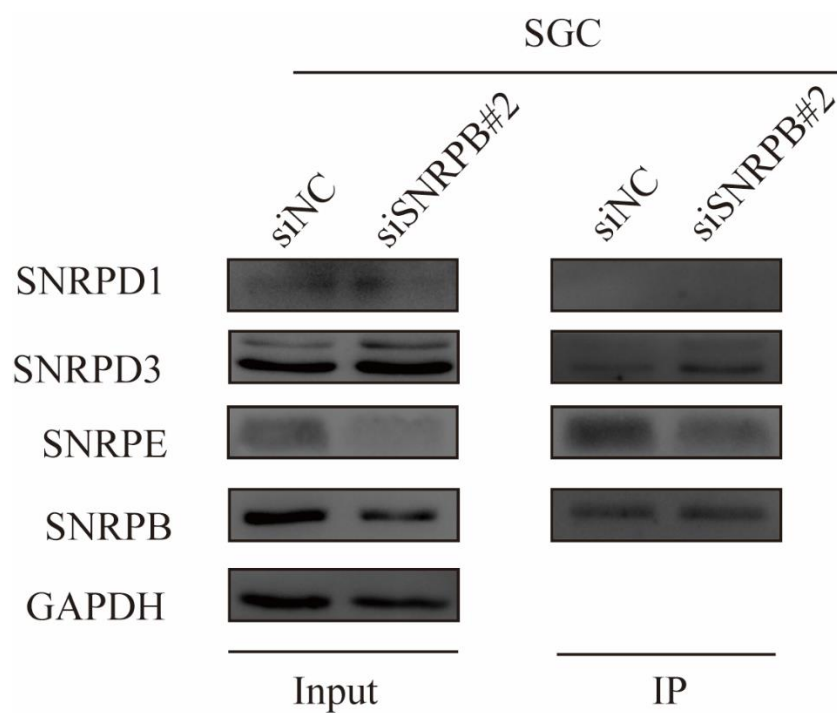

SupplementaryFigure 3

Supplement: Supplementary file 3 — Western Blot original image [file 41419_2025_8011_MOESM3_ESM.pdf]
